# Microvascular network based on the Hilbert curve for nutrient transport in thick tissue

**DOI:** 10.1093/rb/rbae094

**Published:** 2024-08-26

**Authors:** Zhenxing Wang, Xuemin Liu, Xuetao Shi, Yingjun Wang

**Affiliations:** National Engineering Research Centre for Tissue Restoration and Reconstruction, South China University of Technology, Guangzhou 510006, China; School of Materials Science and Engineering, South China University of Technology, Guangzhou 510640, China; Key Laboratory of Biomedical Engineering of Guangdong Province, South China University of Technology, Guangzhou 510006, China; Key Laboratory of Biomedical Materials and Engineering of the Ministry of Education, South China University of Technology, Guangzhou 510006, China; Department of Gynecology and Obstetrics, The Third Affiliated Hospital of Guangzhou Medical University, Guangzhou 510150, China; National Engineering Research Centre for Tissue Restoration and Reconstruction, South China University of Technology, Guangzhou 510006, China; School of Materials Science and Engineering, South China University of Technology, Guangzhou 510640, China; Key Laboratory of Biomedical Engineering of Guangdong Province, South China University of Technology, Guangzhou 510006, China; Key Laboratory of Biomedical Materials and Engineering of the Ministry of Education, South China University of Technology, Guangzhou 510006, China; National Engineering Research Centre for Tissue Restoration and Reconstruction, South China University of Technology, Guangzhou 510006, China; School of Materials Science and Engineering, South China University of Technology, Guangzhou 510640, China; Key Laboratory of Biomedical Engineering of Guangdong Province, South China University of Technology, Guangzhou 510006, China; Key Laboratory of Biomedical Materials and Engineering of the Ministry of Education, South China University of Technology, Guangzhou 510006, China

**Keywords:** 3D bioprinting, microvascular networks, thick tissue constructs, 3D cell culture

## Abstract

To address the uneven nutrient distribution within three-dimensional (3D) tissue models and organoids currently used in medical research, this study introduces a microvascular network based on the Hilbert curve. Our aim was to develop innovative solutions for enhancing nutrient supply in thick tissue models *in vitro*. By using 3D bioprinting, we engineered microvascular networks of varying Hilbert orders and validated their efficacy in enhancing nutrient uniformity through numerical simulations and experiments. These networks facilitated broader and more uniform nutrient distribution throughout the thick tissue models, particularly the 2° Hilbert microvascular structure, which occupies less space and significantly reduces regions of cellular death. Furthermore, we explored the potential of assembling larger tissue constructs using the 2° Hilbert microvascular network, showcasing its applicability in constructing large-scale biological models. The findings suggest that the 2° Hilbert microvascular structure is particularly effective in ensuring adequate nutrient delivery, thus enhancing the viability and functionality of large-volume tissue models. These innovations hold significant promise for advancing the fields of tissue engineering and regenerative medicine by improving nutrient delivery to *in vitro* thick tissue block models. This provides a robust foundation for future *in vitro* research and clinical applications, potentially leading to more effective treatments and interventions in the medical field. The development of these microvascular networks represents a crucial step forward in overcoming the limitations of current 3D tissue models and organoids, paving the way for more sophisticated and reliable biomedical research tools.

## Graphical abstract

**Figure:**
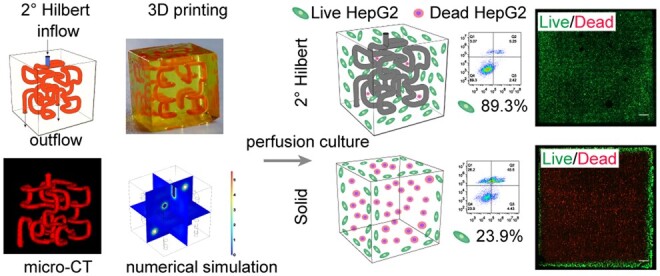

## Introduction

Tissue engineering has emerged as a pivotal field in medical research [1–3], offering promising avenues for addressing complex health challenges. Of particular significance is the *in vitro* cultivation of three-dimensional (3D) tissue models and organ-like constructs [3–5], which serve as invaluable tools for studying disease mechanisms, conducting drug testing and advancing regenerative medicine [6–8]. However, despite their immense potential, these models face a significant hurdle: the challenge of ensuring uniform nutrient delivery to all cells within larger constructs [9]. Traditional static culture methods, which rely on diffusion from the outer surface inward, are inadequate for thicker tissues, which limits model size, cell viability and structural complexity [10–12].

This limitation highlights a critical issue: static cultures fail to mimic the natural efficiency and uniformity of the vascular system in the context of nutrient delivery, and this shortcoming is particularly evident in large-scale, densely cellular tissue structures, where nutrient requirements are higher [7, 13–15]. In recent years, several studies have explored the construction of *in vitro* microvascular networks. Methods such as 3D printing of hydrogel structures with simple perfusion channels [16] and coaxial extrusion for creating functional vascular structures have been investigated [17]. However, these simplified vascular channels often fall short in terms of distribution uniformity and efficiency when used for cell culture, potentially leading to uneven nutrient supply. Additionally, other research efforts have focused on combining pipeline design with microvascular self-assembly to enhance nutrient delivery [18, 19]. Nonetheless, these approaches face challenges such as prolonged cultivation periods, limited choices of materials and cell systems and issues with uncontrollable and irreproducible outcomes. More importantly, the effectiveness of these methods in larger-scale cell culture systems requires further validation [18, 19]. The trade-off between maintaining a substantial proportion of viable internal structures and achieving optimal nutrient delivery remains unresolved.

In this article, we exploit the property of the Hilbert curve to linearly traverse every discrete unit of 3D space. Combined with the diffusion mechanics of nutrients in hydrogels, we selected appropriate Hilbert orders and constructed embedded microvascular network models in thick tissue blocks by using 3D bioprinting. Following long-term perfusion culture, a substantial population of cells within the thick tissue block models remained viable.

Through this design, an efficient and uniform nutrient supply can be achieved in large-volume *in vitro* 3D culture models and organoid models, addressing issues of uneven nutrient distribution and size limitations inherent in traditional techniques. Furthermore, this enables the exploration of larger-volume and more complex 3D tissue models and organoids for studying disease mechanisms, drug testing and applications in regenerative medicine.

## Materials and methods

### Materials

The bioink consisted of 8 wt% gelatin methacryloyl (GelMA), 2 wt% polyethylene glycol diacrylate (PEGDA, 6 kDa), 0.5 wt% lithium phenyl-2,4,6-trimethyl benzoyl phosphonate and 0.1 wt% tartrazine. For the models, the solvents used were phosphate buffered saline (PBS), endothelial cell culture medium for the 2D Hilbert models and PBS containing 0.1 wt% xanthan gum and 15 wt% iodixanol (IDX) for the cell-loaded models.

### Cell culture and maintenance

HepG2 cells were cultured in alpha-minimum essential medium (MEM) supplemented with 10% fetal bovine serum (Sigma‒Aldrich, F0193) at 37°C in a 5% CO_2_ environment and incubated in multilayer cell culture flasks.

### Model design and printing

Models were designed in Blender 3.1 (Blender Foundation, Amsterdam, Netherlands). The order of the Hilbert curves was drawn manually. To simplify the research question, the channel dimensions were uniformly designed to be 750 μm. The channels had a circular cross-section and were created by sweeping the Hilbert curve trajectory through the solid, resulting in a Boolean difference. The printing layer height was set to 50 μm, with a light source wavelength of 405 nm and a light power density of 20 mW/cm^2^. Each layer was exposed for 15 s.

### Imaging and micro-CT

To visualize the internal microchannel architecture of each Hilbert model, red microfil (Flow Tech Inc., Carver, MA, USA) was infused into the channels until they were filled. After microfil solidification within the channels, images were captured using a Sony a6300 camera (Sony, Tokyo, Japan) with a SIGMA Art 70 mm macro lens under RAW settings and lighting provided by a Godox SL60 W. Micro-CT was employed to assess the print fidelity due to the X-ray-opaque nature of the microfil. Scanning was conducted with a Quantum GX2 micro-CT scanner (Revvity) at 88 µA and 90 kV for 4 min, with a 60 µm voxel size resolution and rotation around the microfil-filled models. The CT images were analyzed using Analysis 14.0 software.

### Glucose diffusion coefficients in the hydrogels

To determine the diffusion coefficient of glucose through the hydrogel used in this study, two rectangular diffusion pools were used. Each pool was constructed from two acrylic resin chambers of equal volume, referred to as the donor and receiver phases. The pools were put together by securing a hydrogel sheet between the half chambers and fastening them firmly into a rubber gasket. Each chamber contained 41 mL of PBS, and the donor phase also contained a glucose solution. The solutions were equilibrated at 37°C for 60 min before assembly. The diffusion of glucose in the hydrogel was calculated as follows [20]:

$$V_{d}\frac{\partial C_{d}}{\partial t}=-D_{e}A\frac{C_{d}-C_{r}}{l}$$

where *l* is the thickness of the hydrogel membrane, *A* is the area of the hydrogel membrane, *D_e_* is the effective diffusion coefficient of the hydrogel and *V_d_* is the volume of the donor. *C_d_* is the concentration of glucose in the donor, and *C_r_* is the concentration of glucose in the receiver.

### Numerical simulation

To visualize the spatial and temporal distribution of glucose within each Hilbert model, numerical simulations were conducted using COMSOL Multiphysics. By using species transport and reaction engineering modules, we modeled glucose diffusion in cubes with embedded 1° to 3° Hilbert microvascular networks. The initial glucose concentration inside the models was set to zero, with a constant glucose and oxygen concentration of 5.56 mol/m^3^ in the channels, which were replenished continuously with fresh complete MEM. The reaction rate for glucose consumption by cells was defined as *r* = *k*•*c*, where *r* is the reaction rate, *k* is the reaction constant and *c* is the concentration of the substance.

### Perfusion culture system

The perfusion system consisted of a peristaltic pump (Kamoer, KXP100-GB), a storage bottle, and the model, which were connected with silicone tubing and connectors to form a recirculating perfusion pathway. The storage bottle cap was fitted with a 0.22 µm air filter to allow sterile gas exchange. A custom holder was used to stabilize the connection between the model and the fittings, with the model embedded securely in the holder. The entire assembly was operated inside a 37°C incubator with 5% CO_2_.

### Endothelialization of the channels

To fill the microchannel network, 300–600 µl of human umbilical vein endothelial cell (HUVEC) suspension (1.5 × 10^7^ cells/mL) was infused from the inlet of the 2D Hilbert model. The model entrances were temporarily sealed with clamps. The model was kept at 37°C to ensure that the cells adhered to the channel walls in a nonflow state. After 1 h, the model was turned 180° to allow cell attachment to the opposite side of the channel, resulting in circumferential seeding of cells along the channel walls. Finally, the cell suspension was slowly removed, and the model was incubated at 37°C for an additional 5 h overnight.

### Immunofluorescence staining

Immunofluorescence staining and confocal imaging were utilized to examine the vascular tissue in the 2D Hilbert model. The model was initially washed with PBS for 3 min. Then, 4% paraformaldehyde fixation fluid was infused to fill the internal microchannels. The model was removed from the holder and soaked in 4% paraformaldehyde for 5 min. It was then washed in PBS for 10 min and incubated for 1 h in a blocking solution composed of 5 wt% bovine serum albumin (BSA) and 0.3 wt% Triton X-100 for membrane permeabilization. The model was incubated overnight with primary antibodies in PBS containing 1 wt% BSA. The cells were washed in PBS containing 1 wt% BSA for 10 min to remove unbound primary antibodies. The model and secondary antibodies were incubated in PBS containing 1 wt% BSA for 2 h. Immunofluorescence staining for CD31 was performed using a rabbit polyclonal antibody (CD31, 1:200 dilution, Proteintech, 11265-1-AP). Immunofluorescence staining for von Willebrand Factor (vWF) was conducted using a rabbit polyclonal antibody (vWF, 1:100 dilution, Proteintech, 27186-1-AP). Immunofluorescence staining for VE-cadherin was performed using a mouse monoclonal VE-cadherin antibody (1:200 dilution, Proteintech, 66804-1-Ig). Additionally, the cell nucleus and cytoskeleton were stained with 4',6-diamidino-2-phenylindole (DAPI, Solarbio, C0060) and Actin-Tracker Red-Rhodamine (Beyotime, C2207S), respectively, for cell identification and morphological analysis. A Zeiss LSM 980 confocal microscope with Zeiss ZEN 3.7 software was used with objectives ranging from 10× to 40× using 405, 488, 514, 561 and 633 nm lasers.

### FITC-dextran permeability testing

To identify the barrier function of the printed vascular system, the quantification of fluorescein Isothiocyanate (FITC)-labeled 70-kDa dextran (FITC-Dex; Aladdin, FF478322) permeability was conducted by perfusing the live vascular channels with endothelial cell culture medium containing 25 μg/mL FITC-Dex at a rate of 100 μl/min for 6 min. Fluorescence images were captured every 60 s before and after the start of perfusion, which continued for 6 min, using a Zeiss LSM 980 confocal microscope. The permeability coefficient for FITC-Dex was calculated using the following formula [21]:

$$P_{d}=\frac{1}{I_{1}-I_{b}}(\frac{I_{2}-I_{1}}{t})\frac{d}{4}$$

where *P_d_* is the permeability coefficient, *I*_1_ denotes the mean intensity at the initial time point and *I*_2_ represents the mean intensity at a subsequent time point. *I_b_* refers to the background intensity prior to the introduction of FITC-Dex, and *d* symbolizes the channel diameter. Measurements were conducted for channels with and without an endothelial cell lining (*n* = 3).

### Photorheological characterization

Using an optical attachment with a wavelength of 405 nm and a 20 mm diameter plate, a rheometer (Discovery HR-2, TA, USA) was used to analyze the photorheological parameters of the hydrogels. After dropping bioink onto the bottom plate (with or without cells), the top testing plate was lowered to create a 50 μm gap. A 50-s scan was then performed at an angular frequency of 5 rad/s and a strain of 10%. The sample was pretreated for 30 s and then exposed to 405 nm light for 20 s at a power density of ∼20 mW/cm^2^.

### Distribution of cells in the ink

To facilitate observation of the distribution of cells in the ink after being stationary for at least 1 h, HepG2 cells were stained with DiD (Beyotime, C1039). Initially, DiD was incorporated to the complete MEM at a concentration of 10 μM, and the cells were incubated at 37°C for 20 min. The medium containing DiD was then removed, and the cells were washed twice with Dulbecco's phosphate buffered saline (DPBS). The cells were then digested with trypsin, and the resulting cell suspension was prepared at 8 × 10^6^ cells/mL in bioink. The components of the two types of bioink were the same, except that one contained 0.1% xanthan gum and the other did not. The bioink was added to a cylindrical mold (diameter = 5 mm, height = 9 mm), and after the mold was filled, it was irradiated with 405 nm light for 60 s to solidify the bioink. The mold was then cut in half along the diameter of the bottom face and transferred to a Zeiss LSM 980 confocal microscope for fluorescence observation.

### Printing resolution of the bioink

Three types of bioinks (cell-free, cell-loaded and cell-loaded with IDX) were used to print the same model under the same printing parameters. After construction, the models were observed under a Zeiss LSM 980 confocal microscope in bright field with Tiles mode.

### Live/dead cell staining

Live/dead cell staining was performed using a calcein acetoxymethyl ester/propidium iodide (calcein-AM/PI) assay kit (Beyotime, C2015L) to distinguish between live and dead cells. First, the model was split along the midline and washed with PBS to remove impurities from the culture medium. Then, calcein AM and PI were mixed with PBS at concentrations of 2 μL/mL and 3 μL/mL, respectively, and the model cross-sections were submerged in these solutions and incubated in the dark for 30 min. After incubation, the excess dye was removed by washing with PBS, and the model was immediately observed under a confocal microscope to assess cell viability within the model.

### *In vitro* biocompatibility

The bioink was dispensed into a 24-well plate (1 mL per well) and then crosslinked to the hydrogels using 405 nm blue light irradiation. L929 cells were seeded on the surface of the hydrogel samples at a density of 1 × 10^4^ cells per well and incubated at 37°C in a 5% CO_2_ environment. Sampling was conducted on days 1, 3 and 7, followed by CCK8 cell proliferation assays and live/dead cell staining.

### Flow cytometry

To quantify the live cells in each model, flow cytometry was conducted. First, the model was lysed using 0.6 mg/mL GelMA lysis solution (EFL, GM-LS-001), followed by centrifugation at 1000 rpm for 5 min, after which the supernatant was discarded. The cells were then resuspended in trypsin and incubated at room temperature for 2 min before digestion was stopped by the addition of complete MEM. After another 5 min of centrifugation at 1000 rpm, the supernatant was discarded, and the cells were resuspended in PBS. Approximately 100 000 resuspended cells were centrifuged at 1000 rpm for 5 min, and the supernatant was discarded. The cells were resuspended in 195 μl of Annexin V-FITC binding solution, 5 μl was added, and the solution was gently mixed. The mixture was gently stirred again before adding 10 μl of propidium iodide staining solution. The cells were cultured at room temperature for 15 min before being examined with a flow cytometer (Beckman, CytExpert).

### Liver function testing

The albumin content was determined using the bromocresol green method, in which bromocresol green binds to albumin to form a green complex. The absorbance of this complex was measured at 620 nm using a spectrophotometer to determine the concentration of albumin. The urea content was measured using the UV-glutamate dehydrogenase method. In this method, urease catalyzes the conversion of urea to ammonia, which reacts with α-ketoglutarate and consumes nicotinamide adenine dinucleotide (NADH). The reduction of NADH at a wavelength of 340 nm was determined as an indirect measure of urea content.

### Statistical analysis

All experiments were performed at least three times. The data are presented as the mean ± standard deviation (SD) (*n* ≥ 3) and were analyzed using GraphPad Prism 8 software (ns indicates *P* > 0.05, * indicates *P* ≤ 0.05, ** indicates *P* ≤ 0.01, *** indicates *P* ≤ 0.001, and **** indicates *P* ≤ 0.0001).

## Results and discussion

### Construction of Hilbert models and nutrient transport simulation

**Figure 1.**
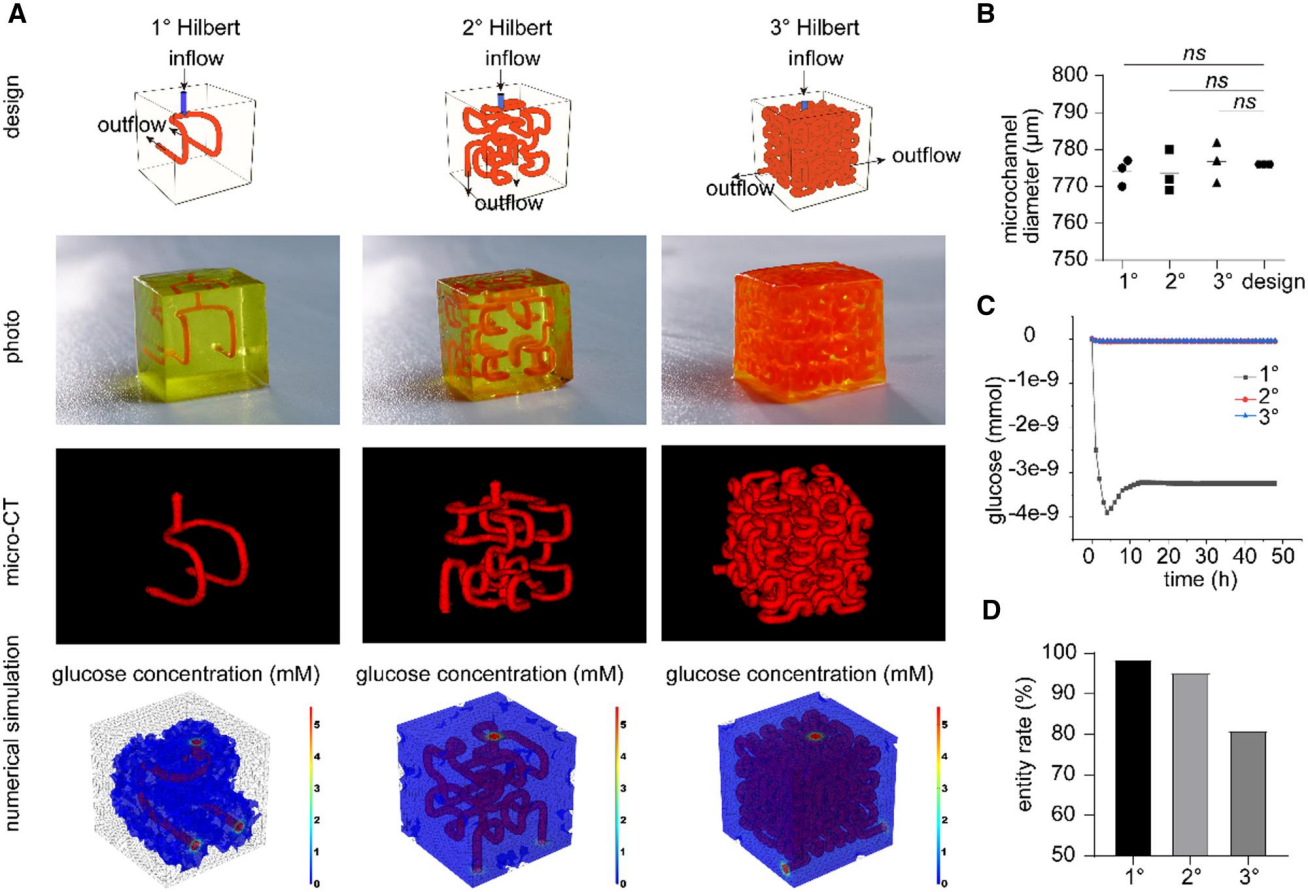
Design and construction of the model with an embedded Hilbert microvascular network. (**A**) Schematic of the model design, printed model (during perfusion, the medium flows into the model from a single port on the upper side and exits from dual ports on the lower side), micro-CT scanning and reconstruction and numerical simulation of glucose nutrient transport in the model. (**B**) Comparison of microchannel diameters in each model measured using micro-CT versus the designed diameters (*n* = 3, *ns P* > 0.05). (**C**) Numerical calculation of the volume fraction of negative glucose concentration regions over time in the 1°–3° Hilbert models. (**D**) Porosity of the 1°–3° Hilbert models. The entity rate is defined as the percentage of the model matrix volume relative to the total volume of the cube.

To determine the appropriate order of Hilbert microvascular networks that allow efficient and uniform nutrient transport in large-volume models, we first used bioink in digital light processing (DLP) 3D-printed embedded Hilbert cubic models ranging from 1° to 3°. To reduce the infusion pressure, the inlet was positioned at half the length of each Hilbert curve, with the topology of the Hilbert curves determining the two outlets of each model. To visualize the microvasculature of each order, the channels were filled with the red non-diffusive dye microfil, which was subsequently solidified and photographed as a physical model (Figure 1A). Due to the X-ray-opaque components of the microfil, micro-CT was used to study the fidelity of the printed structures. The results indicated that DLP 3D technology could faithfully construct cubic models with embedded Hilbert microvascular networks of various orders (Figure 1B). Before constructing the cell-loaded cubic models, we conducted preliminary numerical simulations of nutrient transport through the 1° to 3° Hilbert vascular networks using COMSOL Multiphysics, selecting glucose as the transport substance. The diffusion coefficient of glucose within the hydrogel formed from the bioink was measured using a diffusion chamber and was determined to be ∼5.1 × 10^−10^ m^2^/s at 37°C (Supplementary Figure S1A), and the data were input into COMSOL Multiphysics for computational analysis. As shown in Figure 1A and Supplementary Figure S1B–D, the concentration distribution of glucose 48 h after perfusion in the models of each order was visualized, and grid cells with negative glucose concentrations (i.e. starvation zones or dead zones) were hidden. The cubic model with an embedded 1° Hilbert microvascular network exhibited numerous dead zones, with positive glucose concentrations only near the microvascular network. In contrast, the cubic models with embedded 2° and 3° Hilbert vascular networks had few dead zones, with most areas showing positive glucose concentrations. This demonstrates that compared to the 1° Hilbert microvascular network, the 2° Hilbert and 3° Hilbert networks provide broader and more sufficient glucose transport. The numerical calculations of the volume fraction of areas with negative glucose concentrations over time further corroborate this finding (Figure 1C). Additionally, we calculated the entity rates of the cubic models with embedded 1° to 3° Hilbert vascular networks and showed that the entity rate gradually decreased with increasing order, reducing the space available for cell growth in subsequent experiments (Figure 1D). We conducted *in vitro* biocompatibility tests on the bioink, and the results indicated that L929 cells can maintain long-term viability and exhibit significant proliferation on the surface of hydrogels formed from photocrosslinked bioink (2 wt% PEGDA + 8 wt% GelMA) (Supplementary Figure S2).

### Endothelialization of microchannels

**Figure 2.**
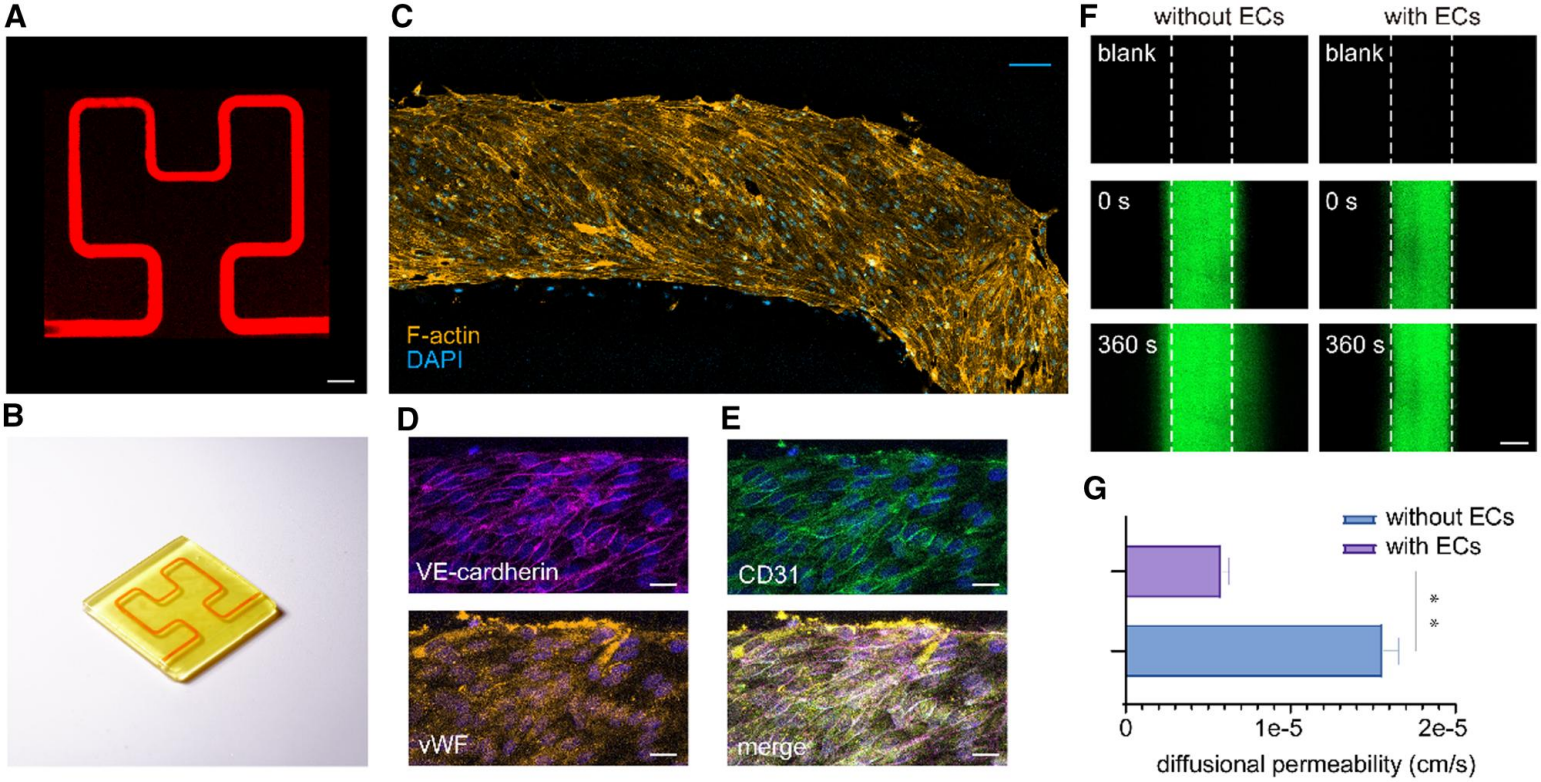
Vascularized microchannels. (**A**) Fluorescence images of the channels in a 2D Hilbert microtube model (scale bar = 1 mm). (**B**) Printed 3D Hilbert microtube model. (**C**) Nuclei and cytoskeleton of endothelial cells on the microtube walls (scale bar = 100 μm). (**D**) VE-cadherin and vWF (scale bar = 20 μm). (**E**) CD31 and DAPI (scale bar = 20 μm). (**F**) Fluorescence images of 70 kDa FITC-Dex diffusion in the microtubes (scale bar = 300 μm). (**G**) Diffusion rate statistics for endothelial cell-lined microtubes and microtubes without endothelial cells (*n* = 3, independent sample *t* test, **P ≤ 0.01).

The endothelialized layer in the vasculature has an important impact on mass transport, cell migration and immune response, etc. The construction of an endothelialized vessel wall in engineered channels is crucial for the study of disease models, drug screening and even the functional realization of artificial organs [22]. To illustrate the establishment of a stable microvasculature, we utilized 3D printing to fabricate a simple channel structure using the same bioink, with the channel’s midline following a 2° 2D Hilbert curve (chamber radius of 750 µm), yielding a model with a circular cross-section 600 µm in diameter (Figure 2A and B). We injected a 5 wt% rhodamine-GelMA solution into the channel and captured fluorescence images with a laser confocal microscope to confirm channel patency (Figure 2A). To clearly visualize the embedding of the channel within the model, red microfil was injected into the channel, and an image of the model was captured (Figure 2B). The channel walls were lined with HUVECs, and endothelial cell culture medium (Sciencecell Cat #1001) was perfused, resulting in the formation of a fused monolayer of endothelial cells arranged along the channel walls (Figure 2C). This microvascular model was perfused in a 37°C incubator with 5% CO_2_ (Supplementary Figure S3), and the culture medium was replaced every 2 days. Notably, after 1 week of perfusion culture, these endothelial cells preserved their endothelial phenotype and demonstrated fusion, as evidenced by the expression of CD31, von Willebrand factor and VE-cadherin, as depicted in Figure 2D and E. To verify the barrier function of the endothelial cells, we perfused a microvascular model with 70 kDa FITC-Dex after 7 days of culture [21], as qualitatively shown in the fluorescence images in Figure 2F. Almost no permeation of the large molecule (70 kDa FITC-dextran) occurred over a 6-min period, with the endothelial cells on the microchannel walls forming a barrier to solute transfer from the lumen to the matrix. This behavior contrasted sharply with that of channels without an endothelial lining. The quantitative results showed that the diffusion permeability of microchannels with an endothelial layer was ∼2.7 times lower than that of microchannels without endothelial cells (Figure 2G). The measured diffusion permeability rate for microchannels with endothelial cells was 5.87 × 10^−6^ cm/s (Figure 2G), similar to the results of other studies reporting the diffusion permeability of *in vitro* formed endothelial monolayers [for FITC-albumin, between O(10^−6^) and O(10^−5^) cm/s] [23, 24] and isolated mammalian small veins (for FITC-albumin, ∼2 × 10^−6^ cm/s) [25]. These results indicate the endothelialization potential of our engineered channels, which provides an advantage for the subsequent construction of vascularized tissue model blocks.

### High-cell density printing

**Figure 3.**
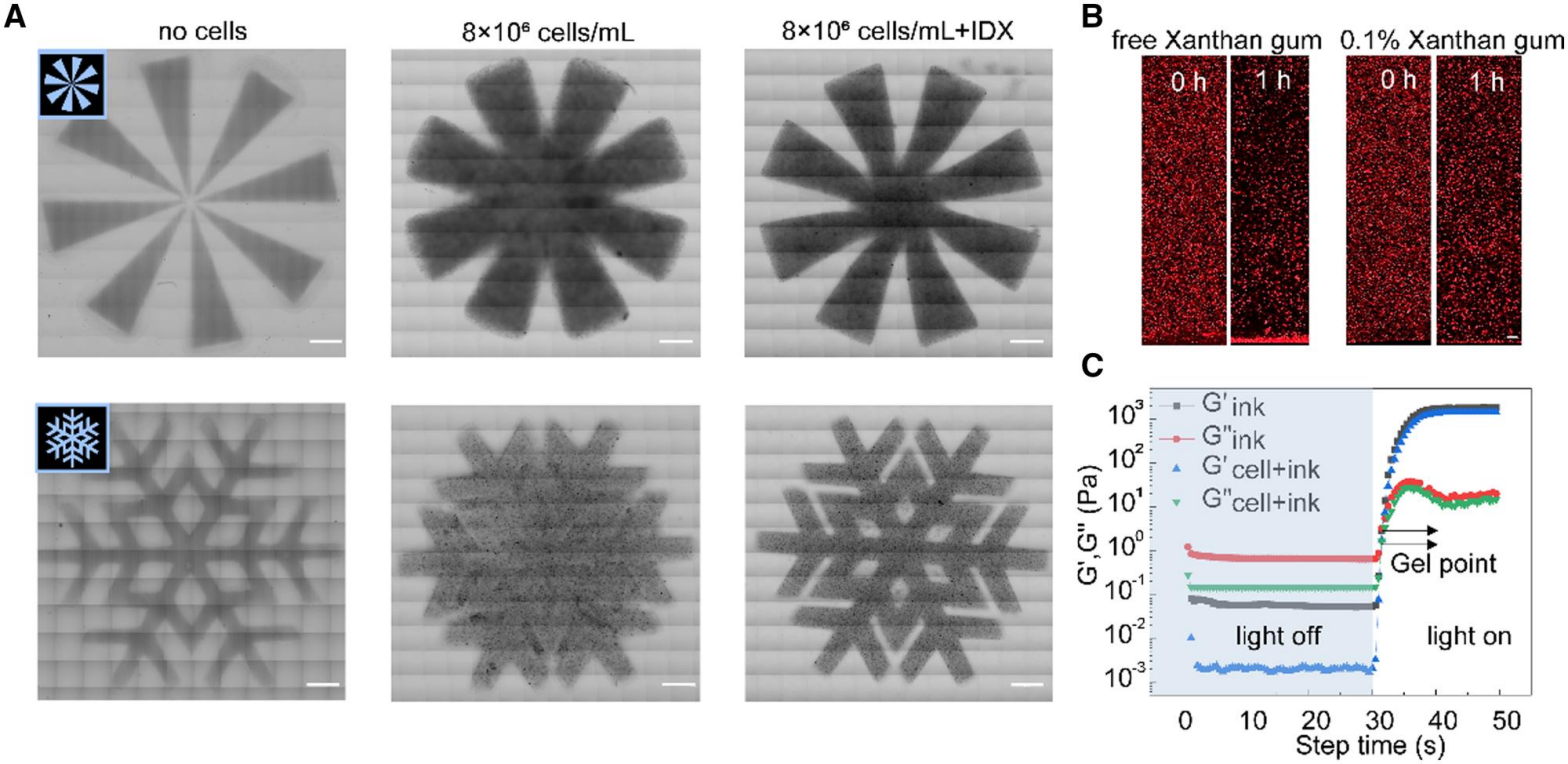
Modulation of bioink performance. (**A**) Print resolution comparison among three different bioink formulations: cell-free bioink, bioink containing 8 × 10^6^ cells/mL and IDX-containing bioink with 8 × 10^6^ cells/mL (scale bar = 1 mm). (**B**) Time-based cell distribution comparison between xanthan gum-free cell-loaded bioink and xanthan gum-containing cell-loaded bioink (scale bar = 200 μm). (**C**) Gelation time comparison between cell-free bioink and cell-loaded bioink.

The demand for microvascular nutrient transport is more urgent in high-cell density models than in traditional *in vitro* 3D cell culture models with a lower cell density (approximately 1 × 10^4^–1 × 10^5^ cells/mL). Additionally, high-cell density 3D cell culture models or organ-like constructs more closely resemble the true cell density of human tissues and are more conducive to simulating tissue or organ function [26–28]. Our 3D Hilbert models aimed to achieve a cell density of 8 × 10^6^ cells/mL. However, when the cell density in the bioink was high, the light projection patterns of the DLP 3D printer were strongly scattered by the bioink, significantly reducing the printing resolution. As shown in Figure 3A, the cell-free bioink could faithfully print the designed snowflake and octadecagonal patterns with sharp edges and clear details. In contrast, the high-cell density bioink (HepG2 cell density of 8 × 10^6^ cells/mL) printed under the same parameters produced constructs with blurred contours, fused areas and missing details. To mitigate light scattering caused by the high-cell density bioink, we adjusted the refractive index of the bioink by adding biocompatible iodixanol. As shown in Figure 3A, the high-cell density bioink with 15% IDX could better reproduce the designed patterns, although the details were not as sharp as those of the cell-free bioink, and the printing quality was significantly improved compared to that of the high-cell density bioink without IDX. This is consistent with the results reported in the literature [29].

Additionally, for the construction of large *in vitro* 3D cell culture models, DLP 3D printing times are generally longer (≥30 min), and cells resuspended in aqueous solution after digestion are in an unstable suspended state and susceptible to settling due to gravity. Therefore, the uniformity of cell dispersion in the ink during long printing processes must be considered. Given that the printing time for the 3D Hilbert models was up to 1 h, we conducted a 1-h static experiment with high-cell density bioink containing 15% IDX (HepG2 cell density of 8 × 10^6^ cells/mL). As shown in Figure 3B, initially, the HepG2 cells were evenly dispersed in the bioink. After 1 h of being stationary, the number of cells in the upper and middle layers decreased, while the bottom region showed a marked increase in cell density, indicating that the cells in the bioink settled at the bottom of the ink tank, resulting in an uneven cell distribution as printing progressed. To address the issue of cell settling in the bioink, we added 0.1 wt% natural thickener xanthan gum to the high-cell density bioink containing 15% IDX. As shown in Figure 3B, adding 0.1 wt% xanthan gum to the bioink prevented significant cell settling at the bottom of the ink tank after 1 h of being stationary, maintaining an even distribution of cells in the bioink. This indicates that although the amount of added xanthan gum was low, it could inhibit cell settling in the bioink. Additionally, we characterized the photorheological properties of the cell-free bioink and the high-cell density bioink containing 15% IDX and 0.1 wt% xanthan gum and found that the addition of IDX, xanthan gum, and HepG2 cells did not significantly affect the gelation time of the ink, which was close to that of the initial components of the cell-free bioink (Figure 3C). Therefore, we used the optimized ink formulations to apply in subsequent studies to reduce the influence of unfavorable factors.

### Long-term perfusion culture of thick tissue block models

**Figure 4.**
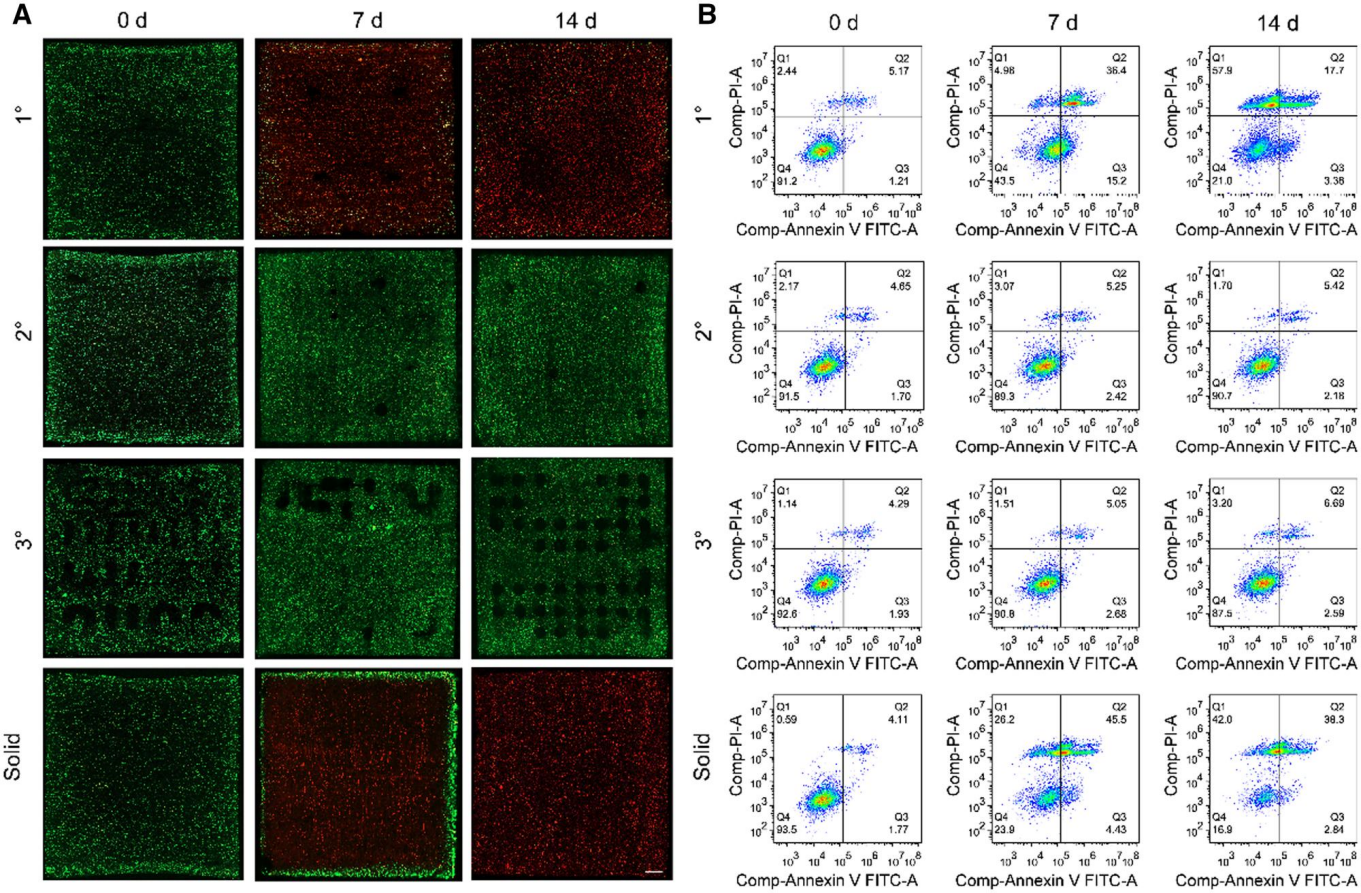
Cell viability assay. (**A**) Fluorescence images of live/dead staining of the central sections of each model at different time points (scale bar = 1 mm). (**B**) Flow cytometry analysis of cells within each model at different time points.

To explore the nutrient transport conditions in the microenvironment of high-cell density microvascular structures, we used a DLP 3D printer to construct thick tissue block models with embedded 1° to 3° Hilbert microvascular networks (dimensions 11*11*11 mm) in a sterile environment, using a similarly sized block without embedded vascular structures as a control group. All the models’ matrices contained HepG2 cells at a cell density of 8 × 10^6^ cells/mL, with the models’ embedded vascular networks lined with HUVECs. The control model was immersed in complete culture medium and cultured statically, while the other models were cultured using a perfusion apparatus. As shown by the live/dead staining results presented in Figure 4A, for all groups, the initial cell viability in the central slice of the models was high, with almost no dead cells visible. Flow cytometry was used to conduct a more comprehensive analysis of cell survival throughout each model (Figure 4B, Supplementary Figure S4), revealing that the initial cell viability in all groups was high, with few dead cells. This suggests that the DLP 3D printing process did not markedly affect cell viability within the models.

For the thick tissue block with an embedded 1° Hilbert microvascular network, after 7 days of perfusion, a large number of HepG2 cells within the matrix died, as determined by flow cytometry, with the cell survival rate decreasing to ∼43.5%. After 14 days of perfusion, the number of live cells in the matrix further decreased, with a cell survival rate of only ∼21.0%. This indicates that the 1° Hilbert microvascular network was unable to provide sufficient nutrients to the HepG2 cells in the matrix of the thick tissue block in a timely manner, causing large areas of the block to become dead zones and preventing long-term survival of the cells in the thick tissue block.

For the thick tissue blocks with embedded 2° and 3° Hilbert microvascular networks, after 7 days of perfusion, fluorescence images of the model centers showed high-cell viability in the matrix, with flow cytometry results showing cell survival rates of ∼89.3% and 90.8%, respectively (Figure 4B). After 14 days of perfusion, the cell viability in the matrix remained high, with flow cytometry results showing cell survival rates of ∼90.7% and 87.5%, respectively (Figure 4B). This indicates that both the 2° and 3° Hilbert microvascular networks can provide broad regional coverage and sufficient nutrients for high-density HepG2 cell growth in thick tissue blocks, maintaining the long-term activity of the thick tissue blocks.

For the control group without an embedded vascular network, after 7 days of culture in culture medium, fluorescence images of the model center showed extensive cell death in the interior regions, with only a ring of cells less than ∼1 mm from the outer surface surviving (Figure 4A), and the flow cytometry results showed that the cell survival rate decreased to ∼23.9% (Figure 4B). After 14 days of culture, very few cells survived in the central slice of the model, with flow cytometry results showing a cell survival rate of only ∼16.9% (Figure 4B). This indicates that for high-cell density thick tissue models, relying solely on nutrient absorption from the outer surface is far from sufficient for long-term cell survival within the model, making it crucial to embed a vascular network to achieve nutrient transport to the interior of the model.

Additionally, by comparing the structures of the 1° to 3° microvascular networks, we also found that for long-term culture in thick tissue models, not only is it necessary to embed a vascular network, but the structure of the embedded vascular network is also crucial for the survival of cells in all regions of the model, as a reasonable vascular structure allows for broader nutrient transport coverage within the space occupied by the thick tissue model, reducing the occurrence of dead zones. For thick tissue blocks of this size (dimensions of 11*11*11 mm), embedding either a 2° or 3° Hilbert microvascular network can maintain long-term cell survival within the blocks. Furthermore, considering that the volume occupied by the 2° Hilbert microvascular network in the thick tissue block is smaller than that in the 3° block, thus allowing a larger interstitial volume for more cells to survive, it is more appropriate to choose the 2° Hilbert microvascular network for nutrient transport in thick tissue blocks of this size (dimensions of 11*11*11 mm).

### Long-term perfusion culture of large-scale thick tissue block models

**Figure 5.**
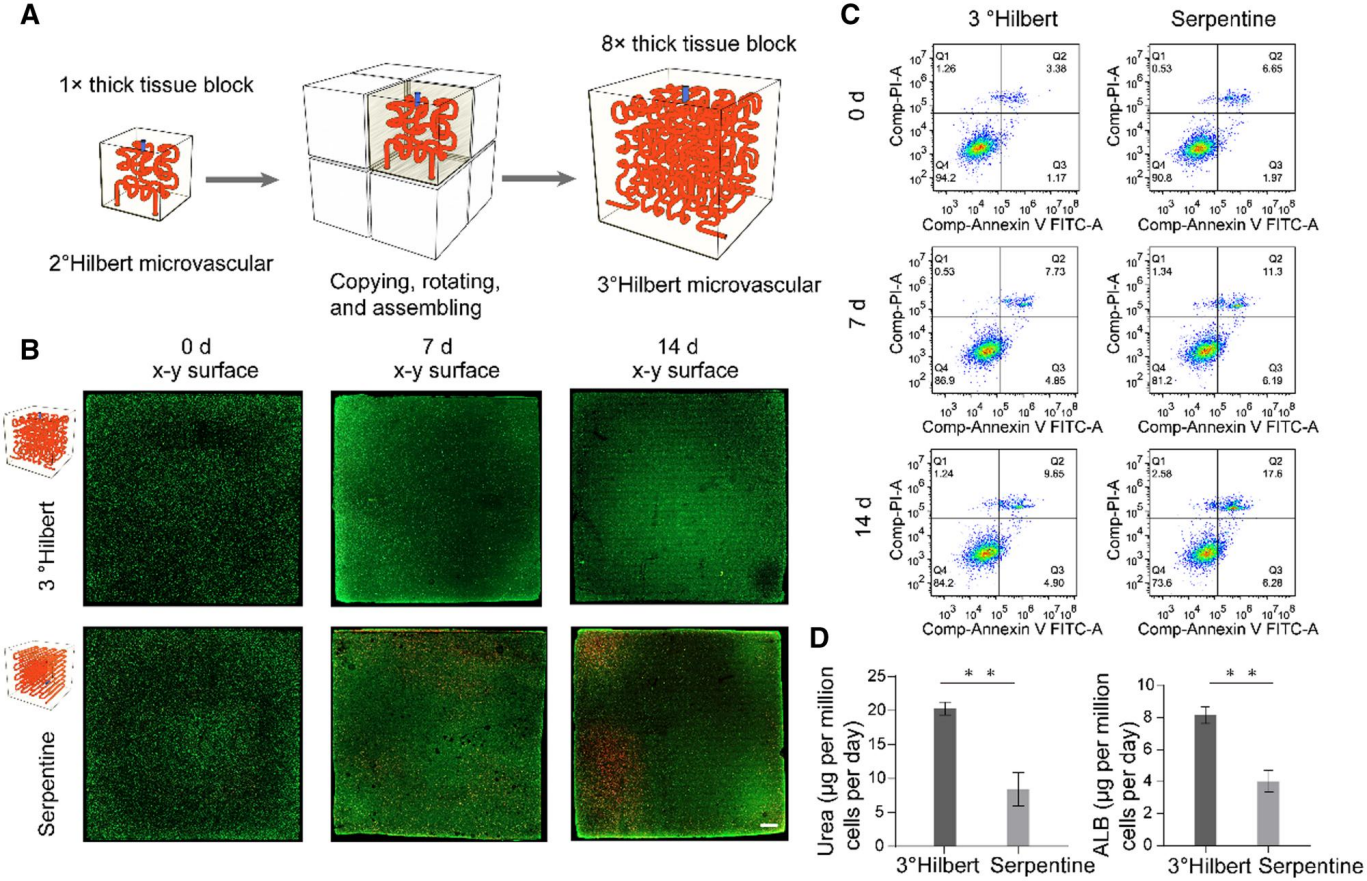
Characterization of 8× thick tissue blocks with an embedded 3° Hilbert microvascular network. (**A**) Schematic diagram of the assembly of 1× thick tissue blocks with embedded 2° Hilbert microvascular networks into 8× thick tissue blocks. (**B**) Fluorescence images after live/dead staining of central sections of thick tissue blocks with an embedded 3° Hilbert microvascular network and serpentine microtubes (scale bar = 2 mm). (**C**) Flow cytometry analysis of cells in each model at different time points. (**D**) Production of urea and albumin in each group (*n* = 3, **P ≤ 0.01).

The Hilbert curve is a type of fractal curve, with each order of the 3D Hilbert curve formed by rotating and connecting eight curves of the previous order [30]. Therefore, we used this characteristic of the Hilbert curve to rotate and assemble eight optimally selected thick tissue blocks with embedded 2° Hilbert microvascular networks (dimensions of 11*11*11 mm) to form a larger volume thick tissue block with an embedded Hilbert microvascular network (essentially a 3° Hilbert microvascular network) (dimensions of 22*22*22 mm, as the volume is eight times that of the initial thick tissue block, which we refer to as an 8× thick tissue block with an embedded Hilbert microvascular network) (Figure 5A). We constructed an 8× thick tissue block using cell-loaded bioink (HepG2 cells, 8 × 10^6^ cells/mL) and perfused it, establishing a thick tissue block with an embedded serpentine vascular network as a control group (the length of the serpentine vascular network was equal to that of the 3° Hilbert vascular network, with an equivalent wall area). The spacing of serpentine curves is similarly set based on the depth of penetration, with priority given to the design needs of the center of the structure.

As shown by the live/dead staining results presented in Figure 5B and Supplementary Figure S5 for these two groups, the initial cell viability within the thick tissue blocks was high, with almost no dead cells visible. The flow cytometry results indicated that cell viability in both groups was greater than 90%. This indicates that even when the construction time of the thick tissue blocks with embedded 2° Hilbert microvascular networks doubled, the DLP 3D printing process still had little impact on cell viability within the models. This also suggests that our system (including materials, structure and processing method) has the potential to be applied to the construction of large-scale thick tissue blocks.

For the 8× thick tissue block with an embedded Hilbert microvascular network, after 7 days of perfusion, fluorescence images of both the model center and the outer surface showed high-cell viability in the matrix (Figure 5B, Supplementary Figure S5A). The flow cytometry results showed a cell survival rate of ∼86.9% (Figure 5C). After 14 days of perfusion, cell viability in the matrix remained high (Figure 5B, Supplementary Figure S5A), with flow cytometry showing a cell survival rate of ∼84.2% (Figure 5C). This indicates that the 3° Hilbert microvascular network embedded in the 8× thick tissue block (formed by combining eight 2° Hilbert microvascular networks) has the same effect as the 2° Hilbert microvascular network embedded in the 1× thick tissue block, providing broad regional coverage and sufficient nutrients for HepG2 cells at high-cell density in the thick tissue blocks and maintaining the long-term activity of the thick tissue blocks.

For the thick tissue block with an embedded serpentine microvascular network, after 7 days of perfusion, the fluorescence images of the model center showed high-cell viability, but the fluorescence images of the model’s outer surface showed that the cells at the corners of the model had died (Figure 5B, Supplementary Figure S5B), with flow cytometry showing a cell survival rate of ∼81.2% (Figure 5C). After 14 days of perfusion, the fluorescence images of the model center showed high cell viability, but a large number of cells at the corners of the model had died (Figure 5B, Supplementary Figure S5A), with flow cytometry showing a cell survival rate of ∼73.6%. This indicates that compared to the 3° Hilbert microvascular network embedded in the 8× thick tissue block, the serpentine microvascular network, although also capable of maintaining long-term survival of cells in the model center under the same conditions of channel length and wall area, covers a smaller area of the tissue block, providing insufficient nutrient transport to the corners of the thick tissue block, which leads to the occurrence of dead zones. Extending the length of the serpentine channel is required to achieve uniform nutrient delivery, and the increase in length results in unnecessary waste of space and elevated perfusion pressure, suggesting that the Hilbert curve is a more efficient pathway design. In addition, after 14 days of perfusion, the 8× thick tissue blocks with embedded 3° Hilbert microvascular networks exhibited greater albumin secretion and urea synthesis than did the control group with serpentine microvascular networks (Figure 5D).

## Conclusion

To address the inability of cells in large-scale *in vitro* tissue blocks to uniformly and sufficiently access nutrients, we compared 1° to 3° Hilbert microvascular networks within a defined space (11*11*11 mm), considering both the coverage of spatial nutrient transport and the volume occupied by the microvascular networks. The 2° Hilbert microvascular network was chosen as the optimal vascular network to insert in the thick tissue block. Building on this foundation, we utilized the fractal properties of the Hilbert curve to assemble thick tissue block models with embedded 2° Hilbert microvascular networks as units to produce a larger volume thick tissue block (22*22*22 mm), with the embedded Hilbert microvascular network still able to provide sufficient nutrients throughout the block. The potential limitations of these approaches may become apparent in the cultivation of larger tissue constructs. The growth of pipelines could lead to increased perfusion pressure, causing ruptures at the perfusion inlets of the structures. It may still be necessary to mimic the hierarchical vascular network found *in vivo*, designing graded vascular networks with varying diameters to form parallel pathways that reduce perfusion pressure. In future research, we can use this block-building approach to construct larger volumes of *in vitro* thick tissue blocks, which has great potential for building and maintaining organ-level *in vitro* organ-like constructs, making possible the application of larger-volume, more complex-structured 3D tissue models and organoids for research into disease mechanisms, drug testing, and regenerative medicine. Thus, the Hilbert curve demonstrates an advantage in applying to the planning of vascular channels in large-scale thick organ models (e.g. liver).

## Supplementary Material

rbae094_Supplementary_Data
