# Supplementary material for: Microvascular network based on the Hilbert curve for nutrient transport in thick tissue
Source: Regen Biomater. 2024 Aug 26;11:rbae094. doi: 10.1093/rb/rbae094 (PMC11441758; doi:10.1093/rb/rbae094)
Supplement: rbae094_Supplementary_Data [file rbae094_supplementary_data.docx]

**Microvascular network based on the Hilbert curve for nutrient transport in thick tissue**

**Authors:** Zhenxing Wang^1,2,3,4^, Xuemin Liu^5^, Xuetao Shi^1,2,3,4*^, Yingjun Wang^1,2,3,4*^

**Affiliations:**

^1^National Engineering Research Centre for Tissue Restoration and Reconstruction, South China University of Technology, Guangzhou, 510006, P. R. China.

^2^School of Materials Science and Engineering, South China University of Technology, Guangzhou, 510640, P. R. China.

^3^Key Laboratory of Biomedical Engineering of Guangdong Province, South China University of Technology, Guangzhou, 510006, P. R. China.

^4^Key Laboratory of Biomedical Materials and Engineering of the Ministry of Education, South China University of Technology, Guangzhou, 510006, P. R. China.

^5^Department of Gynecology and Obstetrics, The Third Affiliated Hospital of Guangzhou Medical University, Guangzhou, Guangdong, China.

*Corresponding author. Email: shxt@scut.edu.cn, imwangyj@scut.edu.cn


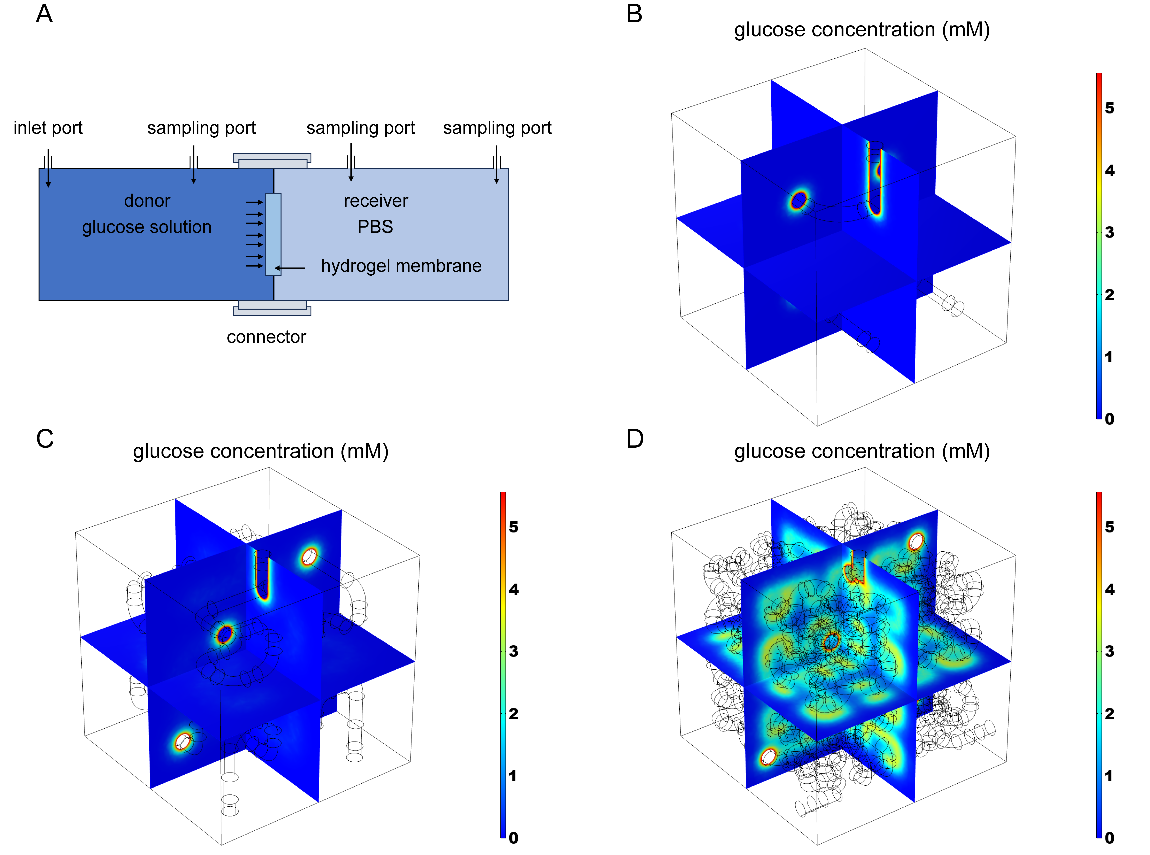


**Fig. S1: Measurement and numerical simulation of glucose diffusion coefficients.**

(A) Schematic diagram of the diffusion coefficient measurements for glucose in the photocrosslinked bioink (hydrogel) using a diffusion device. (B) Multiplane representation of the glucose concentration distribution in a 1° Hilbert model after 48 hours of perfusion. (C) Multiplane representation of the glucose concentration distribution in a 2° Hilbert model after 48 hours of perfusion. (D) Multiplane representation of the glucose concentration distribution in a 3° Hilbert model after 48 hours of perfusion.


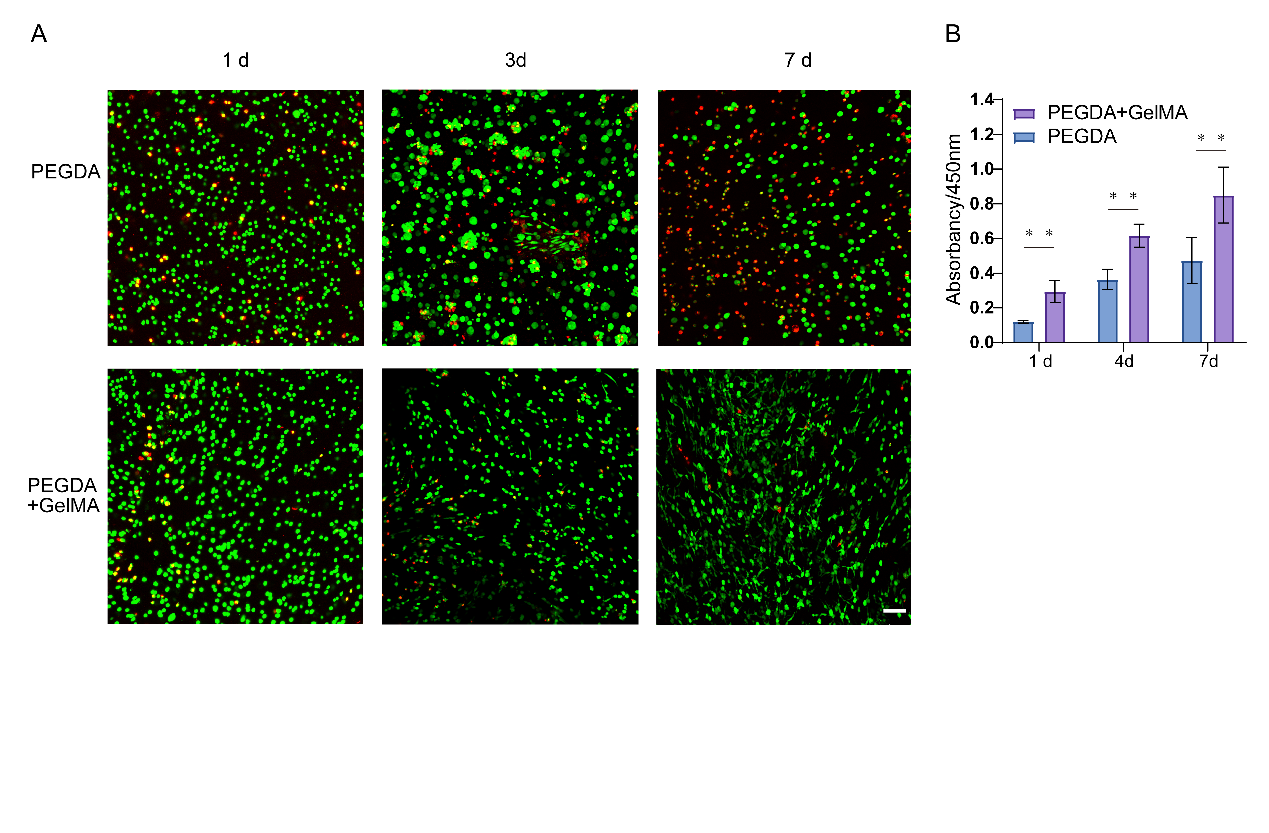


**Fig. S2: In vitro biocompatibility of the photocrosslinked bioink.** (A) Viability of L929 cells on the surface of the photocrosslinked bioink at various time points, characterized by live/dead staining. Control group: 2 wt% PEGDA (6000 Da) hydrogel, with red indicating dead cells and green indicating live cells (red for dead cells, green for live cells; scale bar=100 μm). (B) Cell proliferation of L929 cells in different groups, as assessed by the CCK8 assay (n=3, ** p≦0.01).


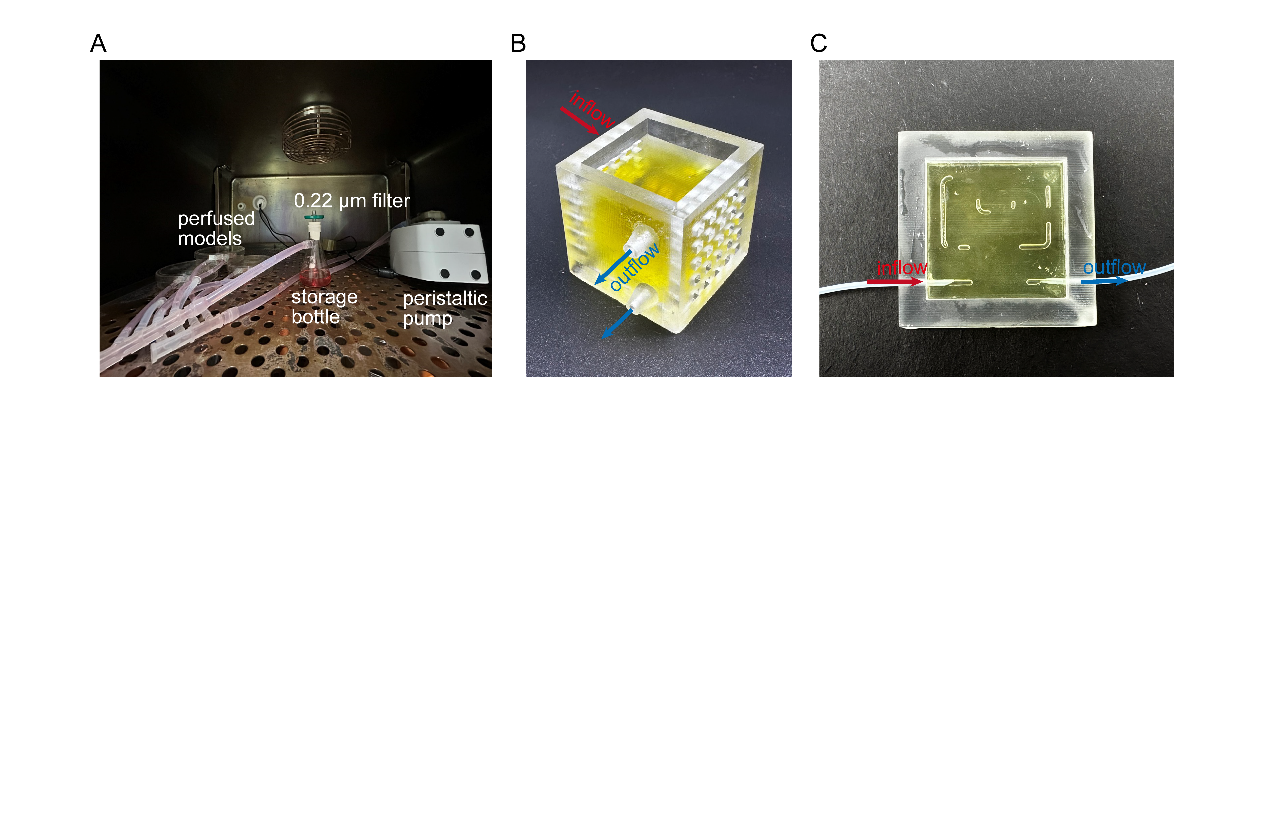


**Fig. S3: Perfusion system.** (A) Main components of the perfusion apparatus. (B) The thick tissue block perfusion model (during perfusion, the medium flows into the model from a single port marked by a red arrow and exits from dual ports marked by blue arrows). (C) 2D Hilbert perfusion model (during perfusion, the medium flows into the model from a single port marked by a red arrow and exits from a port marked by a blue arrow).


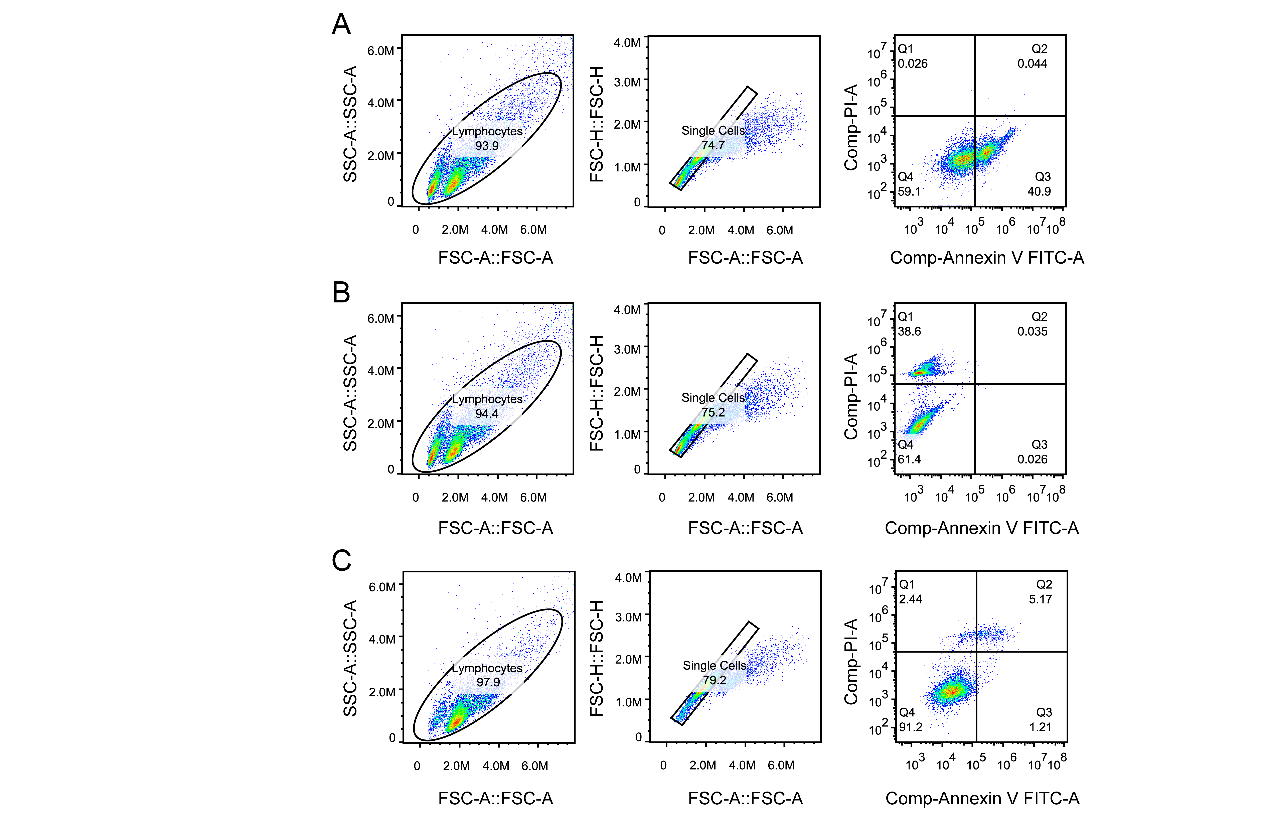


**Fig. S4: Gating of HepG2 cells.** (A) Images of HepG2 cells stained with only Annexin V-FITC. (B) Images of HepG2 cells stained with only PI. (C) Representative flow cytometry (FACS) gating scheme for Annexin V- PI- and Annexin V+ PI+ cells among total HepG2 cells. Annexin V-Pl- indicates viable cells, while Annexin V+Pl+ indicates late apoptotic cells.


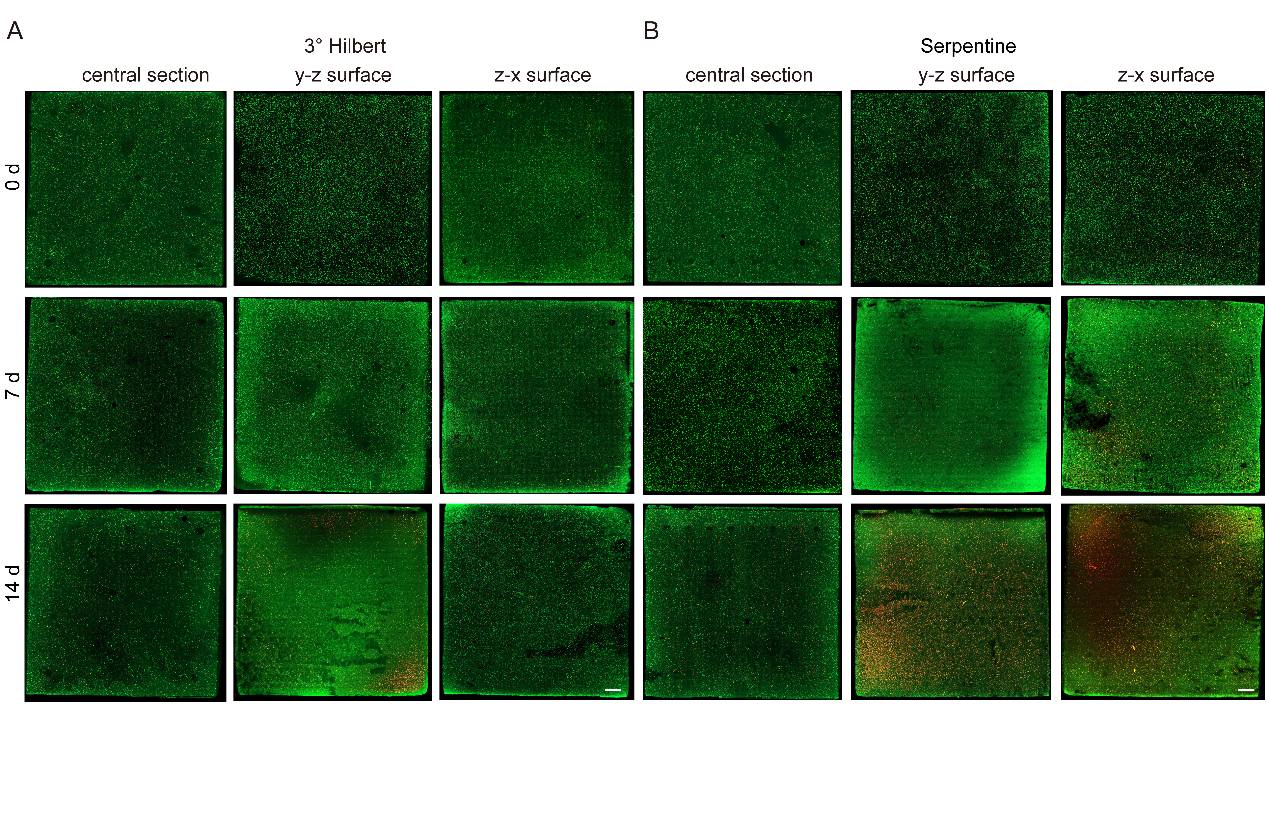


**Fig. S5 Live/dead-stained sections of 8× thick tissue blocks.** (A-B) Fluorescence images obtained after live/dead staining of central sections, the y-z surface and the z-x surface of 3° Hilbert models (A) and serpentine models (B) at different time points (red for dead cells, green for live cells, scale bar=2 mm).
